# Adaptive regulatory substitutions affect multiple stages in the life cycle of the bacteriophage ϕX174

**DOI:** 10.1186/1471-2148-13-66

**Published:** 2013-03-18

**Authors:** Celeste J Brown, Amber D Stancik, Pavitra Roychoudhury, Stephen M Krone

**Affiliations:** 1 Department of Biological Sciences, University of Idaho, Moscow, ID, 83844, USA; 2 Institute for Bioinformatics and Evolutionary Studies, University of Idaho, Moscow, ID, 83844, USA; 3 Department of Mathematics, University of Idaho, Moscow, ID, 83844, USA

## Abstract

**Background:**

Previously, we showed that adaptive substitutions in one of the three promoters of the bacteriophage ϕX174 improved fitness at high-temperature by decreasing transcript levels three- to four-fold. To understand how such an extreme change in gene expression might lead to an almost two-fold increase in fitness at the adaptive temperature, we focused on stages in the life cycle of the phage that occur before and after the initiation of transcription. For both the ancestral strain and two single-substitution strains with down-regulated transcription, we measured seven phenotypic components of fitness (attachment, ejection, eclipse, virion assembly, latent period, lysis rate and burst size) during a single cycle of infection at each of two temperatures. The lower temperature, 37°C, is the optimal temperature at which phages are cultivated in the lab; the higher temperature, 42°C, exerts strong selection and is the condition under which these substitutions arose in evolution experiments. We augmented this study by developing an individual-based stochastic model of this same life cycle to explore potential explanations for our empirical results.

**Results:**

Of the seven fitness parameters, three showed significant differences between strains that carried an adaptive substitution and the ancestor, indicating the presence of pleiotropy in regulatory evolution. 1) Eclipse was longer in the adaptive strains at both the optimal and high-temperature environments. 2) Lysis rate was greater in the adaptive strains at the high temperature. 3) Burst size for the mutants was double that of the ancestor at the high temperature, but half that at the lower temperature. Simulation results suggest that eclipse length and latent period variance can explain differences in burst sizes and fitness between the mutant and ancestral strains.

**Conclusions:**

Down-regulating transcription affects several steps in the phage life cycle, and all of these occur after the initiation of transcription. We attribute the apparent tradeoff between delayed progeny production and faster progeny release to improved host resource utilization at high temperature.

## Background

In a previous study, we showed that adaptive substitutions in one of the three promoters of the bacteriophage ϕX174, which is usually grown in the lab at 37°C, improved fitness at 42°C by down-regulating gene transcription [1]. The down-regulated transcripts overlap with another transcript that we have since shown to be up regulated at 42°C, the adaptive growth temperature [2]. Nonetheless, this result was unexpected because the down-regulated transcripts encode five of six proteins necessary for producing the phage capsid, potentially decreasing the supply of these proteins for progeny production [3]. In addition, these transcripts encode the lysis protein E, which is essential for progeny release. To understand how these substitutions are improving fitness at the adaptive temperature, we have compared the phenotypic components of fitness during a single cycle of infection between strains that carried single adaptive substitutions and the ancestral strain.

The substitutions arose during a series of studies of adaptation to novel environments under conditions in which neutral evolution was minimal [4]. Thirteen experiments were conducted at 37°C and 25 at ≥42°C. In these 38 experiments, adaptive substitutions arose to a detectable frequency a total of 14 times at one of four positions in the −35 σ factor binding site of promoter P_D_ ([5-8]; Wichman pers. comm.). All but two of these P_D_ substitutions arose during growth at temperatures at or above 42°C. At 42°C, strains carrying any of the four P_D_ substitutions have higher fitness than the ancestral strain, as measured by doublings per hour and by their ability to out-compete the ancestral phage in chemostat experiments [1]. At both 37°C and 42°C, each of these four substitutions down-regulates transcription. Because all four adaptive substitutions had the same effect on transcript levels [1], we have studied the phenotypic consequences of down-regulating gene expression by focusing on two of the four substitutions, ^*A*^*323*^*G*^ and ^*C*^*324*^*T*^.

**Figure 1:**
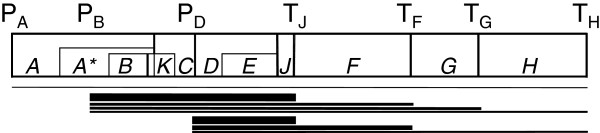
**Genetic map of ϕX174 including relevant sites regulating gene transcription.** Note that the map has been linearized for clarity; ϕX174 has a 5,386 nt circular genome. P_A_, P_B_, P_D_ indicate transcription promoters; T_J_, T_F_, T_G_, T_H_ indicate transcription terminators. *A* to *H* indicate genes, and genes *B, K* and *E* are in different reading frames from the genes with which they overlap. Bars below the map indicate lengths of transcripts found at 37°C. Based on [10,13].

P_D_ promotes the expression of three transcripts (Figure 1) that overlap in their entirety with transcripts produced from an upstream promoter, P_B_[3,9] and are thus redundant. Since the formation of mature capsids requires at least four times more external scaffolding protein D than any of the other phage proteins, P_D_ may be important because it significantly increases the amount of transcript encoding protein D [10]. The lysis protein E is encoded by an out-of-frame gene within the D gene, and changes in transcription from P_D_ may also affect its protein levels. Two of the three transcripts also encode the genes for other capsid proteins, F, G and H. We have recently shown that P_B_ acts like a heat shock promoter at 42°C, increasing the level of transcript for genes B and D, but that the amount of transcript for the capsid proteins F and G is significantly reduced at this temperature [2]. Studies on the bacteriophages λ and T4 have shown that maintaining the proper ratio of capsid proteins is important for maximizing the number of viable phages that are produced per host cell [11,12]. Thus, adaptive substitutions in P_D_ may readjust the capsid protein ratios, and changes affecting transcript and protein levels may be reflected in the phenotypic components of the phage life cycle.

**Figure 2:**
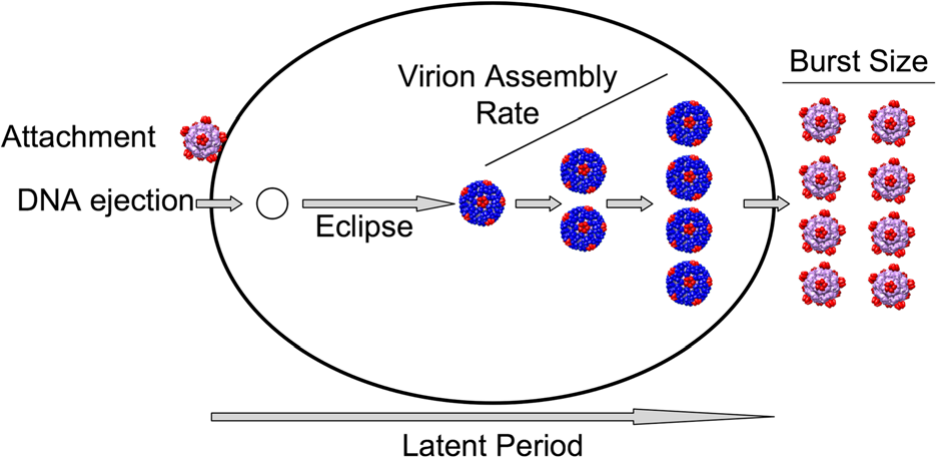
**Diagram of stages in ϕX174 life cycle investigated in this study.** Capsid and virion structures are from PDB accession 1CD3 visualized in Chimera [14].

Each step of the ϕX174 life cycle is fairly well understood (Figure 2), because it has been a model organism for studying viral infection, viral replication, and capsid structure and assembly [10,13]. Attachment is regulated by amino acids in the capsid proteins F, G and H, which interact with lipopolysaccharides on the surface of the bacterial host. Ejection is not fully understood, but the capsid/pilot protein H is instrumental in bringing the circular, single-stranded phage DNA into the host. During eclipse, or the time that it takes once the phage DNA enters the cell until viable virions are produced, the ssDNA is converted to dsDNA, transcription produces mRNA for all of the 11 phage genes (Figure 1), proteins are translated from the mRNA, and rolling circle replication produces more ssDNA that is quickly converted to dsDNA. At the end of eclipse, there are sufficient capsids and DNA maturation protein C so that the ssDNA is packaged into the capsid as it is replicated from the dsDNA. Virion assembly continues until the host cell starts to divide and lysis occurs, thus ending the latent period, the period between ejection and the release of viable phages. Reproductive success can be measured by the number of viable phages produced (burst size) in a single infection cycle although this is not necessarily the whole story in population-level fitness.

To make the connection between individual reproductive success and population level fitness, we developed an individual-based stochastic model that incorporates key aspects of the phage life cycle, including host cell reproduction, viral attachment, eclipse, latent period, lysis rate and burst size. Such models allow us to observe whether the parameters of the life cycle can fully explain the absolute fitness of the phage and to manipulate unobserved parameters to explore potential explanations for our empirical results [15-18].

This study is important, not because it tells us how adaptive regulatory changes affect the fitness of ϕX174 specifically, but because it addresses several questions about the importance of gene regulation in the evolutionary process. First and foremost, we provide a detailed analysis of each step of the life cycle (Figure 2), which includes locating and exploiting resources essential for reproduction (the host cell), progeny production and dispersal. These processes can be generalized to the life cycles of living organisms and have been studied, for instance, in bacteria and yeast. In experiments with microbes in which access to nutrients are manipulated, regulatory changes arise that affect the ability of the microbes to exploit this resource: expression of nutrient transport genes is up regulated at low nutrient levels [19,20], and expression of metabolic operons is down-regulated when the operons are superfluous [21,22]. These regulatory changes lead to improved fitness in the adaptive environment as measured by growth rate and other aspects of organismal physiology [23-25]. Second, bacteriophages are viruses, and this study allows us to observe how a virus adapts to both abiotic (high temperature) and biotic factors (a host cell in heat shock). Simultaneous adaptation to both of these factors may result in tradeoffs between different fitness components [26]. Third, we address the question of whether pleiotropy is universal (i.e., potentially affecting all traits) for regulatory substitutions, or restricted to steps that occur after the initiation of transcription, or specific to a single step in the life cycle. Mutations that have a large positive effect on fitness are expected to affect few phenotypic traits [27,28]. In particular, mutations in cis-regulatory regions are expected to have few pleiotropic effects since they affect only the expression of the genes they regulate [29]. We are only now learning, however, about the pleiotropic effects of genes in general [30,31]. And fourth, using model simulations, we link trait variability at the level of individual phage to average burst size and mean population fitness. We use our model to explore the effects of different life cycle parameters and unobserved traits such as variance in latent period [18,32] on fitness.

To understand how down-regulating transcription might increase fitness at high temperature, we compared each step in the phage life cycle (Figure 2) between the ancestral phage genotype and two strains of phages that differed from the ancestor at a single position each: mut323 with substitution ^*A*^*323*^*G*^ and mut324 with substitution ^*C*^*324*^*T*^. We use parameter estimates of the measurements at these steps in a stochastic cellular automata model that simulates phage population dynamics to determine whether these estimates can predict the differences in fitness that are seen under experimental conditions between the ancestor and the mutant strains. Using the model, we also investigate variance in latent period [32] as determined by the standard deviation of the latent period distribution and its effect on burst size and fitness. We show that this lytic phage adopts strategies that manipulate limited host resources as has been found for other viruses [33,34].

## Results

### Adaptive substitutions do not affect attachment or ejection

**Figure 3:**
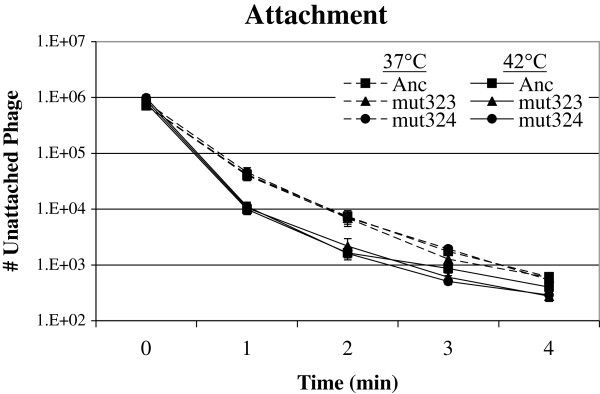
**Mutants do not differ from Ancestor in attachment at either temperature.** Counts of unattached phages (y-axis) vs. time since inoculation of *E. coli* C cultures (x-axis). Error bars indicate ± 1 SE.

In most of our experiments, we pre-attach phages to the cells. Phages in fitness assays and chemostats, however, are not pre-attached to cells. We tested whether there are differences in attachment between the mutants and the ancestor, even though there are no differences in the amino acid sequences of any of the phage proteins involved in attachment. Figure 3 and Table 1 show that although the time at which 50% of the phages are attached to the cells is earlier at 42°C than at 37°C, there are no significant differences among the three strains at each temperature (37°C: F = 0.08, df = 2,15, p > 0.91; 42°C: F = 0.13, df = 2, 15, p > 0.87). Therefore, differences in attachment rates do not explain the differences in fitness among these strains [1].

**Table 1:** Fitness parameters (± 1 st. error) estimated for three phage strains

**Strain/Temp**	**Attachment**^**1**^	**Ejection**^**2**^	**Eclipse**^**3**^	**VA rate**^**4**^	**Latent per.**^**3**^	**Lysis rate**^**5**^	**Burst size**^**6**^	**Fitness**^**7**^
Anc/37C	31.8 ± 0.16	2.2 ± 0.30	8.4 ± 0.10*	4.9 ± 0.2	12.4 ± 0.04	13.6 ± 0.57*	156.2 ± 12*	20.9 ± 0.1*
mut323/37C	31.8 ± 0.12	2.0 ± 0.13	9.8 ± 0.15	5.0 ± 0.2	12.9 ± 0.28	6.6 ± 0.29	79.1 ± 2.7	19.9 ± 0.1
mut324/37C	31.7 ± 0.18	2.0 ± 0.23	9.7 ± 0.15	4.4 ± 0.2	12.5 ± 0.03	8.3 ± 0.38	96.4 ± 3.7	17.9 ± 0.3
Anc/42C	30.4 ± 0.03	1.7 ± 0.16	8.5 ± 0.14*	3.1 ± 0.1	11.2 ± 0.08	1.7 ± 0.12*	17.9 ± 0.7*	9.2 ± 0.2*
mut323/42C	30.3 ± 0.03	1.6 ± 0.34	9.6 ± 0.17	3.3 ± 0.2	11.2 ± 0.21	3.6 ± 0.25	35.9 ± 1.4	16.9 ± 0.4
mut324/42C	30.4 ± 0.04	1.6 ± 0.10	9.4 ± 0.13	3.0 ± 0.2	11.0 ± 0.27	4.1 ± 0.14	43.5 ± 1.1	14.1 ± 0.7

^1^Time (sec) at which 50% of phages growing in phage LB are attached.

^2^Time (min) at which 50% of phages incubated in starvation buffer are ejected.

^3^Earliest time (min) at which the number of phages doubles relative to the previous time point.

^4^Fold increase in intracellular virions per minute.

^5^Virions released per minute.

^6^Number of phages at 24 (37°C) or 22 (42°C) minutes post-infection.

^7^Doublings per hour, from [1].

*Statistically significant difference (p < 0.05) among phages at indicated temperature.

**Figure 4:**
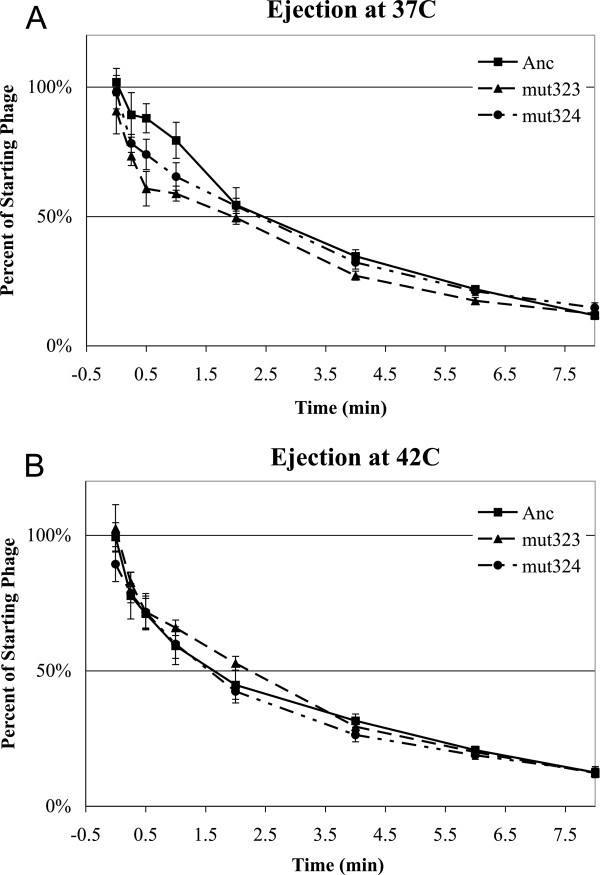
**Mutants do not differ from Ancestor in ejection at A) 37°C or B) 42°C.** Percent of starting phages that have not ejected (y-axis) vs time since the initiation of ejection. Phages were pre-attached to cells at 15°C and ejection was initiated by transferring pre-attached phages to the incubation temperature. Error bars indicate ± 1 SE.

In the next step of the phage life cycle, bacteriophage DNA is ejected into the cell, and in ϕX174 this is facilitated by the pilot protein H. We tested whether there are differences in ejection between the mutants and the ancestor, possibly due to conformational differences in the secondary structure of the ssDNA. Figure 4 and Table 1 show that although the time at which 50% of the phages are ejected into the cells is earlier at 42°C than at 37°C, there are no significant differences among the three strains at each temperature (37°C: F = 0.099, df = 2,13, p > 0.90; 42°C: F = 0.27, df = 2, 13, p > 0.76). Therefore, differences in ejection rates do not explain the differences in fitness. Note that this assay was undertaken under extreme growth conditions, and although it reflects the relative differences among the phage strains, it should not be taken as a measure of the ejection rate in rich media such as phage LB.

### Adaptive substitutions affect length of eclipse but not virion assembly

**Figure 5:**
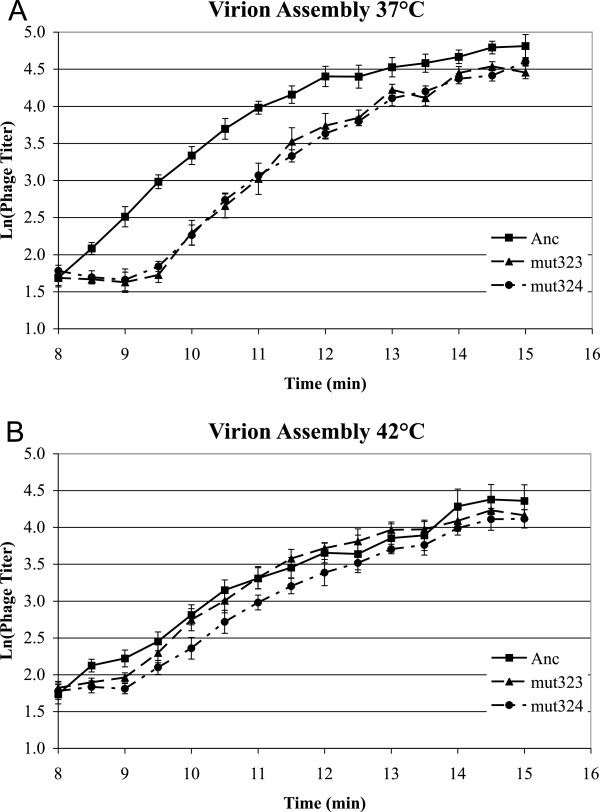
**Mutants differ from Ancestor in eclipse time but not virion assembly rate at both A) 37°C or B) 42°C.** Number of intracellular phages (y-axis) vs. time since the initiation of ejection into *slyD* cells (x-axis). Phages were pre-attached to cells at 15°C, unattached phages were removed and ejection was initiated by transferring pre-attached phages to the incubation temperature. Cells were lysed to release intracellular phages; *slyD* cells are not lysed naturally by ϕX174. Phage count was the same at earlier time points as at the 8 min time point. Error bars indicate ± 1 SE.

The time it takes from ejection until viable virions are found inside the cell is the eclipse period. The increase in viable phages from the end of eclipse to cell lysis is the rate of virion assembly. To observe the length of eclipse and the rate of virion assembly, we used a strain of *E. coli* that carries a mutation in the slyD protein that blocks host cell lysis by the phage lysis protein E. Figure 5 and Table 1 show that although the eclipse period does not differ between the two temperatures, in the ancestor eclipse ends sooner than in the mutants at each temperature (ANCOVA, strain: 37°C: F = 36.9, df = 2, 14, p < 0.0001; 42°C: F = 6.35, df = 2,13, p = 0.01). However, virion assembly is much faster at 37°C than at 42°C, but the difference among the strains is not significant at each temperature (ANCOVA, time*strain interaction: 37°C: F = 0.37, df = 2,123, p > 0.7; 42°C: F = 0.82, df = 2,124, p > 0.43). Thus, lower transcript levels of the mutant strains at both temperatures are delaying the start of genome packaging into capsid, but not the rate at which packaging proceeds.

### Adaptive substitutions do not affect latent period but do affect lysis rate and burst size at both temperatures and fitness at 42°C

**Figure 6:**
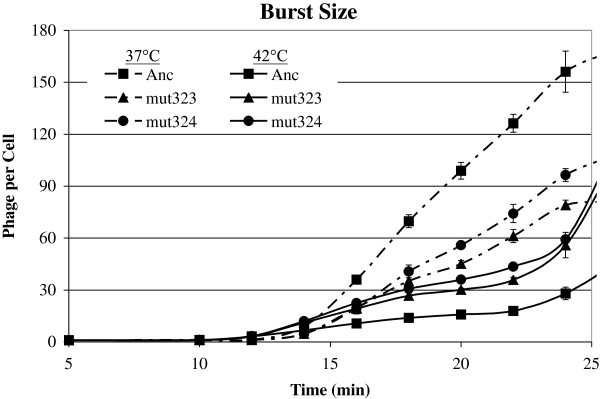
**Mutants do not differ from Ancestor in latent period but differ significantly in burst size at both temperatures.** Mutants have lower burst size at **A**) 37°C and greater burst size at **B**) 42°C. Number of extracellular phages (y-axis) vs. time since the initiation of ejection (x-axis). Error bars indicate ± 1 SE.

The point at which the phage begin to lyse the cells defines the end of the latent period. This can be considered the earliest point in time at which new phages are produced in the culture, and we define this as the earliest time at which the number of phages doubles relative to the preceding time point. Figure 6 and Table 1 show that contrary to our previous results, which used a slightly different protocol [1], there was no difference in latent period among the strains at each temperature (37°C: F = 1.5, df = 2,6, p = 0.29; 42°C: F = 0.2, df = 2,7, p = 0.82). There was a large difference among the strains in burst size. By 24 min the ancestor produced more phages per cell at 37°C (F = 30.2, df = 2,6, p < 0.001) and by 22 min produced fewer phages/cell at 42°C (F = 181.8, df = 2,7, p < 0.0001) than the two mutant strains. Thus the lysis rate (traditionally called the rise), which we define as the increase in the number of released phages over time and is indicated by the slope of the burst assay curve, is greater for the ancestor at 37°C (time*phage interaction: F = 2.58, df =12,41, p = 0.012) and greater for the mutants at 42°C (time*phage interaction: F = 3.69, df =14, 49, p = 0.0003). These results show that the low transcript levels of the mutant strains have a negative effect on reproductive success at 37°C and a positive effect on reproductive success at 42°C.

### Model simulations show that differences in eclipse and variance in latent period may explain fitness differences between Anc and Muts

We developed an individual-based stochastic model of the phage life cycle and parameterized the model with empirical estimates of host and phage life cycle events. We used simulations of this model to examine the extent to which the phenotypic changes we observed could explain differences in burst sizes and fitness among the strains. Because mut323 and mut324 had similar parameter estimates, we made a composite Muts strain for the simulations.

*Differences in length of eclipse alone cannot explain differences in burst size*: We started with a very simple simulation to ask: all else being equal, how does a difference in length of eclipse affect burst size? Simulations were initialized with 50 infected cells and run for ~9 × 10^5^ steps, which corresponds to 25 min, the typical length of a latent period/burst size assay. To prevent noise due to a second round of infection, no uninfected cells were placed on the grid. Based on experimental values at 37°C (Table 1), start of lysis was set to 12.5 min; end of lysis to 24 min and eclipse was set to 8.5 min for the ancestor (Anc) and 9.8 min for the mutants (Muts). In these simulations, we assumed that Anc and Muts produce progeny phages at the same rate, as suggested by the viral assembly assay. The phage population at the end of the simulation was divided by the starting number of phages to obtain an estimate of the productivity per starting phage, similar to the burst size assays (Table 2). As expected, we found that longer eclipse results in lower productivity or mean burst size (Welch’s t = 71, df = 2.0, p < 0.001) but this simulation with a difference in length of eclipse alone does not explain the two-fold difference in burst size between Anc and Muts in the experiments (Table 1).

**Table 2:** Simulation parameters and predicted mean burst size and fitness (± 1 st. error)

**Strain/Temp**	**Host GR**^**1**^	**Eclipse**	**VA rate**^**2**^	**LP Min**^**3**^	**LP Max**^**4**^	**LP SD**^**5**^	**Burst size**^**6**^	**Fitness**^**7**^
Anc/37C	1.9	8.5	16.7	12.5	24	0	162.9 ± 0.03	NA
Muts/37C	1.9	9.8	16.7	12.5	24	0	140.3 ± 0.3	NA
Anc/37C	1.9	8.5	16.7	12.5	24	1	151.7 ± 2.0	20.5 ± 0.1
Muts/37C	1.9	9.8	16.7	12.5	24	4	89.1 ± 4.2	18.4 ± 0.1
Anc/42C	2.4	8.5	6.0	11	22	7	26.8 ± 1.1	14.6 ± 0.5
Muts/42C	2.4	9.5	6.0	11	22	1	38.7 ± 0.4	15.6 ± 0.1

^1^Host growth rate, doublings/hour.

^2^Virion assembly rate.

^3^Minimum length of latent period.

^4^Maximum length of latent period.

^5^Standard deviation of the latent period distribution (min).

^6^Average number of phages produced in a single infection cycle.

^7^Predicted fitness after multiple rounds of infection, doublings/hour.

**Figure 7:**
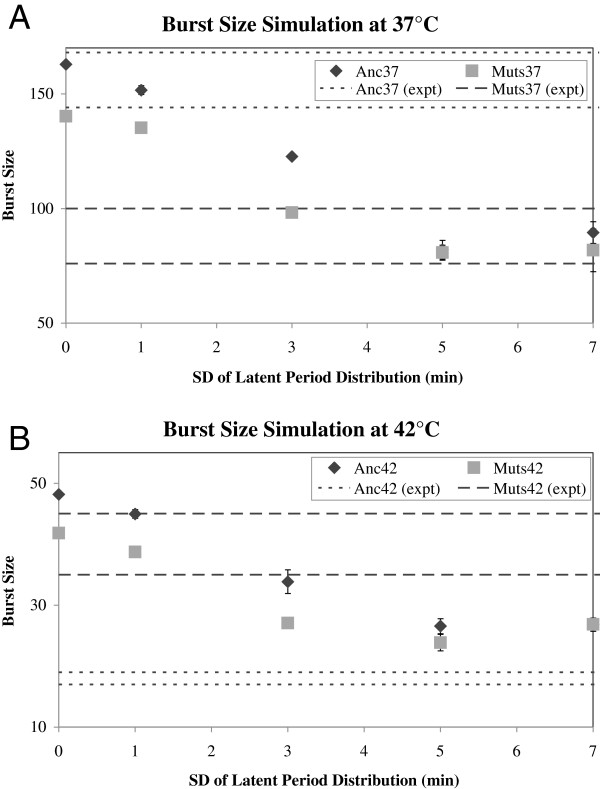
**Increasing variance in latent period decreases expected burst size.** Simulation results showing effect of increasing variance in latent period on expected burst size for 37°C (**A**) and 42°C (**B**). Dotted horizontal lines indicate 1SD intervals for burst sizes from experimental results in Table 1. The burst size or per-phage productivity at the end of the simulation (y-axis) is determined for increasing values of the standard deviations of the latent period in the simulation (x-axis).

*Effect of latent period variance*: Since the difference in eclipse alone did not explain the difference in empirical burst sizes between Anc and Muts, we explored another model parameter that we did not measure directly, but could be added in the simulations. Variance in latent period distribution has been proposed to affect fitness of several phages [18,32]. Lysis rate depends on the timing and size of a large number of burst events, providing a natural source of variability. From the burst assay curves (Figure 6), we observed that the mean times for the start and end of lysis are the same for Anc and Muts, so it may be the variance in the latent period distribution that is responsible for the differences in lysis rate. We examined the effect of variance in latent period by running simulations where the standard deviation of the latent period distribution was varied between 0 and 7 min. All other parameters were retained from the previous simulation (see Methods). Increasing variance leads to more bursts that are very large and very small. However, since the model assumed that there is a limit to the number of progeny that can be produced by a single infected cell, there is a diminishing benefit from the right tail of the latent period distribution. We found that variance in latent period impacts simulation burst sizes significantly with larger variances producing smaller bursts on average, and the empirical burst size for Muts was reached when SD = 4 min (Figure 7A). This suggests that variance in latent period due to lower transcript levels may be another reason for reduced burst sizes of the mutants at 37°C.

*Effect of temperature on burst size*: Metabolic processes inside a cell and host growth rate are faster at 42°C than at 37°C [35], and our results show that this influences the life cycle of the phages. At 42°C, lysis starts 1 min earlier and ends 2 min earlier than at 37°C. On the other hand, viral assembly rate is far slower at 42°C than at 37°C (Table 1) possibly due to reduced capsid stability [36,37]. We asked whether these differences, in addition to differences in eclipse (8.5 min in Anc, 9.5 min in Muts), could explain the difference in burst sizes between Anc and Muts by repeating the previous simulations with these parameters. Once again, differences in eclipse and latent period alone could not explain differences in burst size between Anc and Muts (Figure 7B). In this case, however, increasing the variance in latent period for the ancestor to seven minutes was necessary to attain a burst size as low as ~27 phages per cell. This suggests that higher transcript levels may result in greater variance in latent period in the ancestor at 42°C, a possibility that we discuss below.

*Fitness implications of adaptive substitutions*: We have previously shown that the fitness of the mutant strains as measured by doublings/hour is significantly greater than the ancestor at 42°C [1]. To test whether this fitness increase was due to changes in eclipse time and variance in latent period, we ran simulations using these parameters for the ancestor and mutants at the two temperatures. Based upon the results in Figure 7, Anc was set to have low variance in latent period at 37°C (SD = 1 min) and high variance at 42°C (SD = 7 min), while Muts have high variance at 37°C (SD = 4 min) and low variance at 42°C (SD = 1 min). Fitness was computed in terms of doublings per hour using the phage population at the end of the simulation (Table 2). As expected, fitness was lower at 42°C compared to 37°C for both Anc and Muts, and Muts had lower fitness than the ancestor at 37°C (t = 11, df =3.9, p < 0.001) and higher fitness at 42°C (t = 3, df = 5.8, p < 0.03). Predicted fitness is within 0.5 doublings per hour of the average empirical fitness for all conditions [1].

## Discussion

The adaptive substitutions in P_D_ affect three of the seven life cycle stages that we measured. First, eclipse length is longer in the mutants than in the ancestor at both temperatures, and therefore these mutations have a negative impact on this component of fitness. Second, the rate at which viable phages are released into the culture is faster in the mutants than in the ancestor at the adaptive temperature, 42°C, although the rate is slower in the mutants than the ancestor at the lower temperature, 37°C. Third, the faster lysis rate of the mutants at 42°C overcame the longer eclipse time thus leading to a larger burst size than the ancestor. The combination of later eclipse and greater variance in latent period for the mutants inferred from the simulations led to a smaller burst size than the ancestor at 37°C. The details of how ϕX174 adapts to a novel environment can help us to understand the role of gene regulation in evolution, especially for a virus adapting to its host. It also points to potential pleiotropic effects of regulatory variants and the benefits of using mathematical models to understand empirical data.

### Role of gene regulation in evolution

In many experimental evolution studies, yeast, bacteria and viruses have been grown under strong selective conditions with the goal of understanding adaptation. Selective conditions that are used in these studies are often produced by limiting a nutrient, providing a novel nutrient, or changing environmental conditions such as temperature or salinity. For studies using viruses, a novel host is sometimes the selective condition, and is similar to both providing a novel nutrient and changing environmental conditions. Quite often the response to selection is a change in gene regulation either by gene duplication, transposon integration, or point mutations in promoters or regulatory genes. Regulatory changes have occurred in both directions. When a particular nutrient is limiting, adaptation takes the form of more transport genes or higher enzyme concentrations. When a particular enzymatic pathway is unnecessary, expression of that pathway is reduced.

We have observed the phenotypic effects of an adaptive regulatory change that down-regulates the expression of structural rather than enzymatic proteins. These structural proteins form the capsid of the phage or cause host cell lysis. Decreasing expression of these genes, therefore, might negatively affect reproductive success by decreasing the number of progeny or delaying progeny release. Nonetheless, the mutant phages were more fit than the ancestor at the adaptive temperature of 42°C. Only by understanding the biology of the phage can we understand this seemingly counterintuitive result and the complexity of gene regulation in adaptation.

First, it is apparent that down-regulating expression of the redundant transcripts affects progeny production by delaying the end of eclipse and the start of virion assembly. This is evident from the later time to eclipse of the mutant phages at 37°C and 42°C. For a capsid to form from its constituent subunits, minimum concentrations of those subunits must be present [38,39]. These minimum concentrations are far greater than the numbers of subunits needed to produce a single capsid. With less transcript, the mutants may be reaching the critical concentration of proteins later than the ancestor. The correct ratio of proteins has been shown to be important for efficient capsid assembly in other bacteriophage [11,12]. A single capsid contains 240 D, 60 B, 60 F, 60 G and 12 H subunits. Thus at least four times as many D proteins are required for capsid assembly than any of the other proteins. If the mutants are making less transcript for D, then the concentration of D may be rate limiting, causing a delay in the assembly of capsid. Taken alone, this is clearly a negative consequence and does not explain the greater fitness of the mutants at 42°C.

Second, down-regulation does not affect virion assembly, the next step in the phage life cycle. That the rate of virion assembly does not differ between the ancestor and the mutants at either temperature suggests that capsid assembly is not the rate-limiting step once virion assembly begins. The assembly of viable virions requires more than the assembly of capsid [10]. Phage DNA and the DNA packaging protein J is packaged into the capsid during virion assembly, and this only happens in the presence of the DNA maturation protein C and replicating DNA. Thus the concentrations of protein C, protein J or the replication initiation protein A, or the rate of ssDNA replication may be limiting. Protein J is encoded on transcripts from P_B_ and P_D_, and if its concentration was rate limiting we would expect to see a decrease in virion assembly rate in the mutant strains. The replication initiation protein is encoded on an unstable transcript with a weak promoter, P_A_, which is far upstream of P_D_, and protein C is encoded from transcripts that come from P_B_ (Figure 1). The concentrations of these two proteins should be unrelated to the transcript levels from P_D_ and may be, therefore, the components limiting the rate of virion production. Alternatively, the attachment of proteins A and C to dsDNA leads to replication of ssDNA via the rolling circle method; once this pre-initiation complex is formed in the presence of sufficient capsid, the assembly of virions may proceed at the rate of DNA replication. Whatever the molecular mechanism, the regulatory substitutions do not appear to be affecting this stage of the life cycle. At 37°C the similarity in assembly rates means that the mutants have far fewer progeny at the end of the latent period, but at 42°C they have about the same number (Figure 5).

Third, the stage in the life cycle that was affected by the regulatory substitutions differently at the two temperatures was the rate at which the cells lysed, an essential component of progeny dispersal. A positive effect of the adaptive substitutions was seen at high temperature, with a negative effect at the lower temperature. The lysis rate, which we define as the slope of the regression line for number of progeny over time, depends upon the number of virions in the cell at lysis and how quickly the cells lyse. Lysis is caused by an interaction between phage protein E and the host enzyme phospho-MurNAc-pentapeptide translocase (MraY), which is involved in peptidoglycan biosynthesis of the bacterial cell wall [40]. Protein E inhibits membrane bound MraY activity, so that upon cell division, construction of the septum fails, and phages are released [41]. Thus, the presence of the E protein and the rate at which the host divides determines the lysis rate [42].

At 37°C the negative effect of the mutants on lysis rate might be explained by lower transcript levels for the lysis protein (Figure 1). There were more ancestral phages at the end of the latent period (12.5 min) than mutant phages based upon the virion assembly assays (Figure 5A). Based on our simulations, this difference in intracellular phage progeny did not seem to explain the differences in burst size. However, increasing latent period stochasticity for the Muts improved the model predictions. There may be a minimum number of E proteins that are necessary for lysis. If down-regulation from P_D_ affects the amount of E protein, we might expect that lysis would occur later. Indeed, in our previous experiments at 37°C in which unattached phages were removed prior to infection (reducing variability), the latent period of the mutants was longer than that of the ancestor [1]. In the present study, the minimum latent periods were the same but increasing the latent period variance of the mutants improved the fit of the simulation results to the actual results for burst size. Similar work with phage λ showed that latent period variance could be attributed to a decrease in promoter activity for the holin gene [32,43]; the λ phage holin protein regulates lysis timing as does protein E. We conclude that at 37°C, down-regulating expression of half the transcripts that encode the lysis protein may lead to increased variance in latent period and lower burst size.

At 42°C the faster lysis rate of the mutants might be explained by the timing of host cell division. The number of intracellular phages just prior to lysis appears to be the same in the ancestor and mutants at this temperature based upon the virion assembly assays (Figure 5B). The faster population-level lysis rate of the mutants at 42°C would be determined therefore by how quickly the cells lyse. Phage infection severely slows the growth of *slyD* cells and alters host mRNA levels; unpublished results suggest that the mutant strains have a faster lysis rate at 42°C, because the cells they infect grow faster than cells infected with the ancestral phage (data not shown). The benefit of down-regulating transcription is greater at 42°C because the rates of host replication, transcription and translation machinery are much greater at this temperature than at 37°C [35]. Thus the host may be overwhelmed by the demands of the phage when the metabolic rate is greater, and progression through the host cell cycle slows. Since the mutant strains produce fewer transcripts, more resources for host transcription and translation may be available, thus increasing host growth rate. Recent work with phage λ showed that slower host growth rate led to increased latent period variance, called lysis time stochasticity in [32]. Based on this result, we increased variance in latent period in our simulations of the ancestral strain at 42°C, and found that burst size decreased. We could not increase variance enough to bring the simulated burst size as low as the experimental burst size; a standard deviation of 5.5 overlaps with the minimum and maximum latent period lengths. Therefore the distribution of latent periods may not be normal as we have assumed here, but possibly has a negative skew toward the later time points.

We conclude that at 42°C, down-regulating expression of half the phage transcripts may lead to increased cell growth, decreased variance in latent period and greater burst size. Interestingly, this conclusion is consistent with studies in *E. coli* that the cost of higher than necessary gene expression is in the processes of transcription and translation [21]. In this case, a virus is adopting a similar strategy to one its host uses to improve growth rate.

### Gene regulation and pleiotropy

The extent of pleiotropy in constraining evolution has been an important consideration in developing evolutionary theory, and recent genomic research has attempted to quantify the degree of pleiotropy in various organisms. For an organism with only 11 genes, it might be expected that pleiotropy due to cis-regulatory mutations would be more extensive than in more complex organisms in which homeostasis is highly evolved*.* Indeed, these adaptive substitutions affect the transcripts for six of these 11 genes. However, these single mutations affecting gene transcription have measurable effects upon only a few traits, all of which occur after the initiation of transcription. Thus the pleiotropic effects of these mutations are not universal nor are they restricted to a single phenotype.

In adapting to a novel environment in which both abiotic and biotic factors exert selection, antagonistic pleiotropy between different stages of the life cycle may result in tradeoffs between these stages. For instance, strong selection for short-term thermal stability in the RNA virus ϕ6 led to increased survivorship but decreased reproductive success [26]. In our study, the largest negative effect for our regulatory mutants at both temperatures is the delayed start of virion assembly, the most immediate process that we measured to which the transcripts contribute. The positive effect on fitness at high temperature includes increasing lysis rate, possibly due to increased host cell growth. This positive effect seems to center upon the processes of transcription and/or translation, rather than on other aspects of progeny production such as assembly rate and latent period. Thus there is a tradeoff between producing transcripts for progeny production and optimizing the use of host resources for transcription and/or translation. At high temperatures, the increase in the rate at which progeny are released has a greater influence on fitness than the decrease in progeny production.

### Linking phenotypes to fitness by simulations

At both temperatures, simulations using our stochastic model of the phage life cycle worked well to predict the fitness of the fittest genotype (Tables 1 and 2). The model did not predict the less fit genotype as well. Indeed, even a longer eclipse did not lead to the much lower burst size of the mutants at 37°C (Table 2). The model was adjusted to take variance in latent period into account, and this brought the fitness estimates within 0.5 doublings per hour of the empirical fitnesses. Although our results are consistent with variance in latent period accounting for the observed differences in burst size, further work is needed to test this hypothesis. We have illustrated the benefits of refining models based upon empirical data and using models to suggest hypotheses for future research. In general, this process of progressively manipulating model assumptions and parameters to account for empirical data is an excellent way to generate new hypotheses.

## Conclusions

When Jacob and Monod first described the mechanistic basis for gene regulation, they immediately recognized the importance of gene regulation in evolution [44,45]. They speculated that the fitness of micro-organisms should rely upon the coordinated expression of functionally-related genes and the repression of unnecessary genes so that valuable cellular resources are not overtaxed. We have shown that growth at high temperature alters the expression of functionally-related capsid proteins, up regulating transcripts for some capsid proteins and down-regulating transcripts for others [2]. We have also shown that one avenue for adapting to high temperature is to down-regulate gene expression of some capsid proteins [1]. Here we show that down-regulating transcription affects several fitness components in the phage life cycle, and each of these components occur after the initiation of transcription. We attribute the apparent tradeoff between delayed progeny production and faster progeny release to improved host resource utilization at high temperature. These results support the evolutionary importance of gene regulation first posited by Jacob and Monod.

## Methods

### Phages and host strains

A lab-adapted isolate of ϕX174 was used as the ancestor for these studies (GenBank Accession AF176034). Strains of ϕX174 with single point mutations in the −35 σ factor binding site of the promoter immediately upstream of gene *D* (P_D_) were described in our previous study [1]. Genotypes of all strains were confirmed at the end of the study by sequencing their entire genomes as previously described [1]. The host used for phage propagation was *Escherichia coli C* kindly provided by HA Wichman [5] except in the virion assembly assay. In the assembly assay, a *slyD* mutant of *E. coli* C, kindly provided by BA Fane, was used because of its resistance to protein E mediated lysis allowing us to follow the accumulation of viable phages inside the cell [46,47].

### Media and buffers

All cultures were grown in phage lysogeny broth (10 g NaCl, 10 g Bacto-Tryptone, and 5 g yeast extract per liter; [6]) with 2 mM CaCl_2_ except for the ejection assay cultures [48], which were grown in KC broth (10 g of tryptone (Difco), 5 g of KCl, 1.0 mL of 1 M CaCl_2_ per liter of distilled water). Starvation buffer (SVB) for the ejection assays contained 5 g of KCl, 1 g of NaCl, 1.14 g Trizma HCl (Sigma), 0.33 g Trizma Base (Sigma), 0.1 g of MgSO_4_, and 1 ml of 1 M CaCl_2_ per liter of distilled water. Elution buffer for the ejection assays consisted of 6 mM EDTA, 0.05 M sodium tetraborate saturated with chloroform.

### Attachment assay

Phage attachment time was determined for each strain at 37°C and 42°C. To start, 5×10^5^ phages were added to 10 ml of an exponentially growing culture of *E. coli* C (app. 2 × 10^8^ CFU/ml). At t = 1, 2, 3, and 4 minutes, a 500 μl sample of the culture was passed through a 0.2 μm syringe filter to remove cells. The filtrate was plated to determine the amount of unattached phages. The time at which half of the phages were attached to the cells was determined by interpolation, and differences among strains were tested using a one-way ANOVA for each temperature.

### Ejection assay

The method of Newbold and Sinsheimer [48] was adapted to measure how long it takes for attached phages to eject their DNA into a cell. *E. coli* C was grown for one hour at either 37°C or 42°C in KC broth to approximately 2 × 10^8^ CFU/ml. Ten ml of culture were centrifuged at 2600 g for 5 minutes at 4°C. The pellets were washed with 10 ml of ice-cold starvation buffer (SVB), and centrifuged again in the same manner as before. The pellet was re-suspended in 1 ml of ice cold SVB and placed at 15°C for 15 minutes. Then 2 × 10^9^ phages of each strain were added to their own set of washed cells in a 1:1 ratio, and the samples were returned to 15°C for 15 minutes. Subsequently, 10 μl of this 1:1 mix were diluted into 5 ml of ice cold LB and 5mls of elution buffer and plated with *E. coli* C to estimate the initial amount of viable phages (t = 0). To begin the assay, 100 μl of the pre-attached phage mix were added to 10 mls of SVB already shaking at the appropriate experimental temperature. At 15 s, 30 s, 45 s, 60 s, 90 s, 120 s, 180 s, 240 s, and 300 s, 100 μl of this culture was diluted into 5 ml of iced elution buffer and thoroughly mixed by vortexing. Dilutions for every time point were kept on ice until the end of the experiment, and then plated for phages that have not ejected their DNA into the cell. This method assumes that all the cells are killed by the chloroform in the elution buffer and phages inside the cell are not viable because eclipse has not started. It also assumes that phages that are attached to the cell are released and can form a plaque. The time at which half of the phages were ejected into the cells was determined by interpolation, and differences among strains were tested using a one-way ANOVA for each temperature.

### Time to eclipse and virion assembly rate

To observe growth kinetics within the host, we modified the procedures of Uchiyama *et al.*[49]. The *slyD* strain of *E. coli* C was grown in phage LB for 1 hour at either 37°C, or 50 minutes at 37°C and then 10 minutes at 42°C. A sample of 1 × 10^8^ cells was put on ice for 5–10 minutes. Then 1 × 10^5^ phages were added, and the sample was placed at 15°C for 30 minutes. At the end of 30 minutes the cells were centrifuged at 13,000 rpm for 5 minutes in a chilled tabletop micro-centrifuge. The supernatant with unattached phages was removed, and the pellet re-suspended in 1 ml of ice cold LB broth, which was then added to 9 mls of LB already shaking at the desired experimental temperature. From 8 minutes to 15 minutes, 500 μl was sampled every 30 seconds and added to 50 μl of chloroform and 5 μl of 50 mg/ml lysozyme to lyse the cells, and then placed back on ice until the end of the experiment. After all time-points were taken cellular debris was removed by centrifuging at 13,000 rpm for 5 minutes in a tabletop micro-centrifuge, and the supernatant was transferred to a new tube and plated for the number of viable phages using *E. coli C*. The time at which the number of viable phages first doubled relative to the previous time point (end of eclipse) was determined by interpolation. To test for differences in eclipse time and virion assembly rate among strains, an analysis of covariance was conducted with strains as the treatment factor and time as the covariate separately for each temperature. The primary test of interest in this ANCOVA is the interaction between time and strain indicating a difference in the rate of virion assembly among strains. Because this interaction term was not significant, the ANCOVA was rerun without the interaction to determine whether there was a significant difference among strains in intercept of the lines, which is equivalent to a difference in eclipse time among strains.

### Latent period, burst size and fitness assays

Latent period and burst size were determined in a single assay. *E. coli* C cells were grown at 37°C for the optimum temperature until a concentration of 2 × 10^8^ CFU/ml was reached as determined by OD_600_. For assays at 42°C, cells were grown at 37°C until just before the culture reached 2 × 10^8^ CFU/ml, then the cells were moved to 42°C for 10 minutes to reach 2 × 10^8^ CFU/ml. Then 1 ml of the cell culture was put on ice for 10 minutes, and 1 × 10^4^ phages were added to the cells and placed at 15°C for 1 hour. At the end of 1 hour this phage and cell mix was added to 9 ml of phage LB broth already shaking at the desired experimental temperature of either 37°C or 42°C. At 5, 10, and every two minutes thereafter up to 30 minutes, the culture was plated for phages. The time at which the number of phages first doubled relative to the previous time point (end of latent period) was determined by interpolation, and differences among strains were tested using a one-way analysis of variance for each temperature. Burst size was determined at 24 minutes post ejection for 37°C cultures and 22 minutes for 42°C cultures, and differences were tested using a one-way analysis of variance for each temperature. To test for differences in the number of phages released over time (lysis rate) among strains, an analysis of covariance was conducted separately for each temperature with strains as the treatment factor and time as the covariate. The primary test of interest in this ANCOVA is the interaction between time and strain indicating a difference in the lysis rate among strains.

### Mathematical model and simulations

An individual-based mathematical model was used to simulate phage population dynamics in liquid culture. Simulations take place on a 200 × 200 grid of sites, with spatial structure removed by considering all sites to be neighbors. Each site on the grid can be empty, occupied by a single bacterial host cell, occupied by one or more bacteriophages, or occupied by both host and phages. A host cell can be uninfected and susceptible to infection by phage, infected by one or more phages, or lysed. Phages are classified as “free”, i.e. phages that have not yet infected a host cell, or “replicating”, i.e. phages that have infected a cell and are in the process of replication within a host cell. The state of the host cell and the population of phages at a site change probabilistically according to rates computed from empirically-derived parameter estimates of various life cycle events like attachment, latent period and host cell division. These rates determine the “wait time” at the site or the time taken for the site to change its state, which is a random variable whose mean is equal to the reciprocal of the corresponding rate.

From a pair of randomly chosen sites, if one site contains an uninfected cell and the other contains one or more free phages, the cell becomes infected at a rate that depends on the attachment time of the phages. After a length of time defined as the latent period, the infected cell lyses and progeny phages are scattered randomly across the grid to begin new infection cycles. The burst size, i.e. the total number of phage progeny released during burst, is proportional to the time between eclipse and the latent period so that cells that have been infected longer produce larger bursts, with a maximum burst size of 200. Values for attachment time and eclipse were drawn from normal distributions with mean and variance determined from experimental values. Latent period was drawn from a truncated normal distribution in which the minimum latent period was the time of earliest lysis events, and the maximum value was taken as the time when the burst assay curve started to plateau. The mean of the distribution was taken as the mid-point of these extremes, and the standard deviation was varied between 0 min and 7 min to explore the effect of variance in latent period on fitness.

Uninfected host cells undergo division at a rate that depends on the doubling time of *E. coli* at the desired temperature: 1.9 dbl/hr at 37°C and 2.4 dbl/hr at 42°C. This rate is scaled according to the number of vacant sites on the grid to simulate Monod-like density dependence [50]. For simplicity we assume that infected cells do not undergo normal cell division and can only lyse at the end of the latent period. We also assume the following: infected cells do not lyse during eclipse; phage do not mutate; the host does not become resistant to phage infection; and multiple infection of a single host is negligible.

During a simulation, sites are updated in random order and the simulation is run for a total of 1.44 × 10^6^ steps, which corresponds to a typical 40-minute fitness assay or 9.04 × 10^5^ steps to correspond to a 25 min burst assay. The time scale in the model was calibrated by running a simulation with only uninfected cells and determining the doubling time. This gives us a scale of 2.17 × 10^6^ steps per hour. Cell and phage populations are recorded every 40,000 simulation steps to track population dynamics. Benchmarking tests were also performed to check that the distribution of burst sizes corresponded to experimental values.

We remark that there are several advantages to running the mass-action model on a 2-dimensional grid with global mixing. First, some of our other work with this phage system involves growth on surfaces. The models for those systems are, by necessity, stochastic and spatial. Using the same platform to simulate both settings allows for more direct comparisons between spatial and well-mixed systems. Second, unlike a differential equation model, the grid-based model already incorporates stochasticity (e.g., latent period) in a natural way. We have shown in related models that the limit (4 × 10^4^ for host cells, and order 10^6^– 10^7^ for phage) imposed by the grid size does not affect the dynamics; i.e., one obtains the same quantitative behavior for larger grid sizes.

## Competing interests

The authors declare that they have no competing interests.

## Authors’ contributions

CJB conceived, designed and coordinated the study and drafted the manuscript. ADS carried out the laboratory studies, participated in their design and helped to draft the manuscript. PR designed the mathematical model, carried out the model simulations and helped to draft the manuscript. SMK helped to design the model and to draft the manuscript. All authors read and approved the final manuscript.
